# The Atlanta College of Physicians and Surgeons

**Published:** 1900-05

**Authors:** 


### *EDITORIAL NOTES AND COMMENTS.*

---

The Business office of THE JOURNAL-RECORD is 305, 306 Fitten Building.

The Editorial office is 318-319-320 The Prudential.

Address all Business communications to Dr. M. B. Hutchins, Mgr.

Make remittances payable to THE ATLANTA JOURNAL-RECORD OF MEDICINE.

On matters pertaining to the Editorial and Original Communications address Dr. Bernard Wolff, Atlanta.

Reprints of original articles will be furnished at cost price. Requests for the same should always be made *on the manuscript.*

We will present, post-paid, on request, to each contributor of an original article, twenty (20) marked copies of THE JOURNAL-RECORD containing such article.

---

### THE ATLANTA COLLEGE OF PHYSICIANS AND SURGEONS.

THE annual commencement of the above institution was held in the Grand Opera House on the evening of April 3d. The audience was one which filled the capacity of this immense auditorium. The exercises were interesting, and being interspersed with excellent music made the evening one of much enjoyment. The graduating class was a large one, numbering seventy-one men, and these received their diplomas in sections. The annual oration was delivered by Rev. Charles E. Dowman, President of Emory College. The medals and honors were delivered by the Hon. E. W. Martin. The Atlanta College of Physicians and Surgeons is now the only regular medical college in the city, and the session which has just closed showed a marked increase in the attendance over previous years. Atlanta offers probably the best clinical facilities of any city in the South, with its large city hospital and the outdoor clinics of the college proper. Beginning with the next session this institution will adopt the four-years graded course, following in the wake of the great eastern medical colleges. The prosperous condition of the session just closed is best appreciated by the annual report of the Dean, and we append this as follows:

*Mr. President and Gentlemen:*

The session now closing has been one of much interest to the faculty and of prosperity to the college. This year has shown beyond the shadow of a doubt that the consolidation of the Atlanta and Southern Medical Colleges was a step of much wisdom, and the Atlanta College of Physicians and Surgeons has been successful far beyond the hopes of its most sanguine supporters. The attendance this year has been considerably above the combined attendance in the two original schools.

The endless entanglements and embarrassing contingencies incident to the coalition have been passed successfully through, and the policy now pervading all the connections of the college bespeak a future of high and untrammelled prosperity.

We are now, gentlemen, admittedly at the head of medical education in the South. With an able and harmonious faculty, with the perfect good-will and support of the profession at large, with buildings well equipped in all their departments, and with students representing a majority of the States of the Union, as well as foreign countries, the institution over which you preside, is enjoying a period of unparalleled and gratifying success.

The urgent demands for higher medical education will prompt us to hereafter require attendance upon four courses of lectures preliminary to gradation, and it is our determined purpose to make this a college of medicine second to none in the country.

In attendance upon lectures in the three departments of medicine, dentistry and pharmacy there have been this year 377 men. Two hundred and sixty-five of these have been in the medical department, and seventy-one ask at your hands the degree of Doctor of Medicine; thirty-four have attended the college of pharmacy, and five ask for the degree of Graduate in Pharmacy; seventy-eight have been enrolled in the Dental Department, and of these a number will, at a future date, ask for the degree of Doctor of Dental Surgery.

Of the 265 medical students 186 hail from Georgia; 17 from South Carolina; 14 from Alabama; 12 from North Carolina; 12 from Florida: 4 from Mississippi; 4 from Texas; 3 from Wisconsin; 1 from Massachusetts; 1 from Virginia: 1 from Arkansas; 1 from Tennessee; 1 from Ohio; 1 from Pennsylvania; 1 from California; 1 from Cuba; and 1 from India.

The faculty desire to commend to the public most heartily the class which graduates on this occasion. We have no doubt they will do honor to the profession and reflect credit upon the institution which now sends them forth. Respectfully submitted,

W. S. KENDRICK, Dean of the Faculty.

